# First record of *Rhopalophthalmus longipes* Ii, 1964 from Malaysian waters (Crustacea, Mysida)

**DOI:** 10.3897/zookeys.642.10316

**Published:** 2017-01-03

**Authors:** Hai Siang Tan, B. A. R. Azman

**Affiliations:** 1 School of Environmental and Natural Resource Sciences, Faculty of Science and Technology, Universiti Kebangsaan Malaysia, 43600, UKM Bangi, Selangor, Malaysia; 2 Marine Ecosystem Research Centre (EKOMAR), Faculty of Science and Technology, Universiti Kebangsaan Malaysia, 43600, UKM Bangi, Selangor, Malaysia

**Keywords:** Malaysian waters, Mysidae, new record, Rhopalophthalmus longipes, taxonomy

## Abstract

The marine mysid species *Rhopalophthalmus longipes* Ii, 1964 is reported from Malaysian waters for the first time. Specimens are described and illustrated in detail based on material collected by epibenthic sledge from the seagrass meadows of Pulau Tinggi, Johor. Specimens exhibit a slight difference from Ii’s type material by possessing a rounded process bearing two small protrusions apically near the middle distal end of the third segment of antennal peduncle. In addition, its telson armed with 7-9 moderately strong setae at the lateral margin.

## Introduction

The genus *Rhopalophthalmus* was established in 1906 by Illig in his preliminary report on the Valdivia Expedition with *Rhopalophthalmus flagellipes* as its type species collected from Congo Estuary, Africa. Later in 1910, Hansen allocated another species to this genus, *Rhopalophthalmus egregius* from Bawean Island in the Java Sea, Indonesia, from the Siboga Expedition.

Currently, the genus *Rhopalophthalmus* contains 27 nominal species (Mees 2010). The previous records of the genus *Rhopalophthalmus* in Malaysian waters were restricted to *Rhopalophthalmus egregius*, *Rhopalophthalmus orientalis* and *Rhopalophthalmus hastatus* (Tan et al. 2014).

*Rhopalophthalmus longipes* was first described from Ajiro, Shizuoka Prefecture, Japan by Ii (1964) during the South China Sea expedition that was conducted by the Imperial Fisheries Experimental Station of Japan. It was then found in the adjacent waters of Nansha Islands, the Spratlys (Wang and Liu 1994) and East China Sea (Wang and Liu 1997). In 2011, Hanamura et al. recorded the occurrence of *Rhopalophthalmus longipes* from Amami Island, south-western Japan, south-western part of the South China Sea, and also the western part of Timor Sea. Since Ii’s original description of *Rhopalophthalmus longipes* is relatively brief and literature from different areas noted morphological variability, the present paper provides a full redescription of *Rhopalophthalmus longipes* collected from Malaysian waters.

## Materials and methods

**Figure 1.**
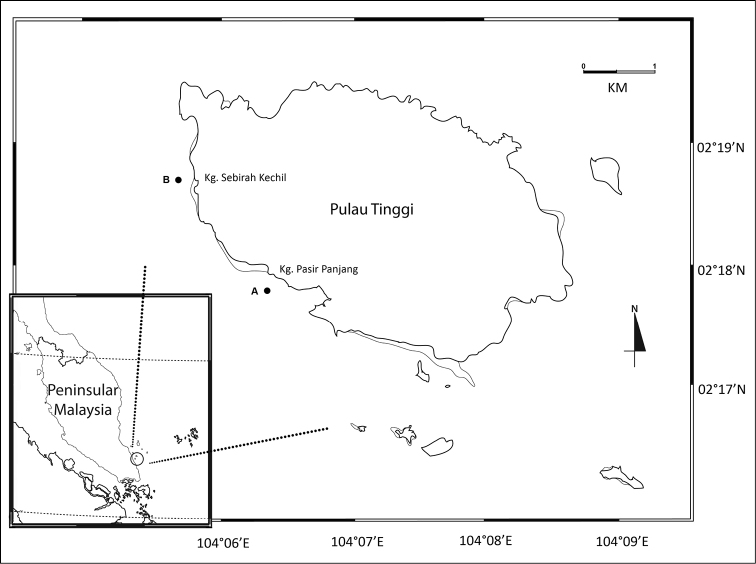
Map of the study area, **A** Kampung Pasir Panjang and **B** Kampung Sebirah Kechil, Pulau Tinggi, Sultan Iskandar Marine Park, Johor.

Specimens were collected from two sites (Kampung Pasir Panjang and Kampung Sebirah Kechil) of seagrass beds from Pulau Tinggi, Sultan Iskandar Marine Park (SIMP), Johor (Fig. 1) by using an epibenthic sledge with a mouth opening of 20 cm height and 60 cm width, mesh size 140 μm. Three replicates were obtained for each station. The appendages and mouthparts were dissected and mounted on glycerol gel slides and then drawn under an optical microscope (Olympus BX43) and stereomicroscope with camera lucida. The drawings were digitized on Adobe Illustrator CC using the methods described in Coleman (2003). Material was deposited in Universiti Kebangsaan Malaysia Muzium Zoologi (UKMMZ).

## Systematics

### Order MYSIDA Boas, 1883 Family MYSIDAE Haworth, 1825 Subfamily RHOPALOPHTHALMINAE Hansen, 1910 Genus *Rhopalophthalmus* Illig, 1906

#### Rhopalophthalmus longipes

Taxon classificationAnimaliaMysidaMysidae

Ii, 1964

Figs 2 , 3 , 4 , 5

Rhopalophthalmus longipes Ii, 1964, 180, figs 46, 47; Mauchline and Murano 1977, 75 [catalogue]; Muller 1993, 49 [catalogue]; Wang and Liu 1994, 91, figs 14; 1997, 204; 2000, 114, figs 27; Hanamura et al. 2011, 14, figs 8-10.

##### Material examined.

One immature female, 6.5 mm, UKMMZ-1553, Kampung Sebirah Kechil, Pulau Tinggi, Sultan Iskandar Marine Park, Johor, 02°18.581'N, 104°05.624'E, epibenthic sledge, 25^th^ March 2012, 30.1 °C, depth 7 m, coll. Azman, B.A.R., Tan, H.S. and Shamsul, B.; eight immature females, six juveniles, UKMMZ-1554; three juveniles, UKMMZ-1555; two immature females, four juveniles, UKMMZ-1556; same station. Largest immature female, 6.9 mm, juveniles, 1.9-3.5 mm, males not collected. In the females, smaller than 5.9 mm, the pleopods are not fully developed. Juveniles: in the smallest specimens at our disposal, measuring 1.9 mm, the eyes are somewhat larger and with stouter stalk than in the adult.

##### Description.

Based on immature female, 6.5 mm, UKMMZ-1553, Figs 2A, 5A.

**Figure 2.**
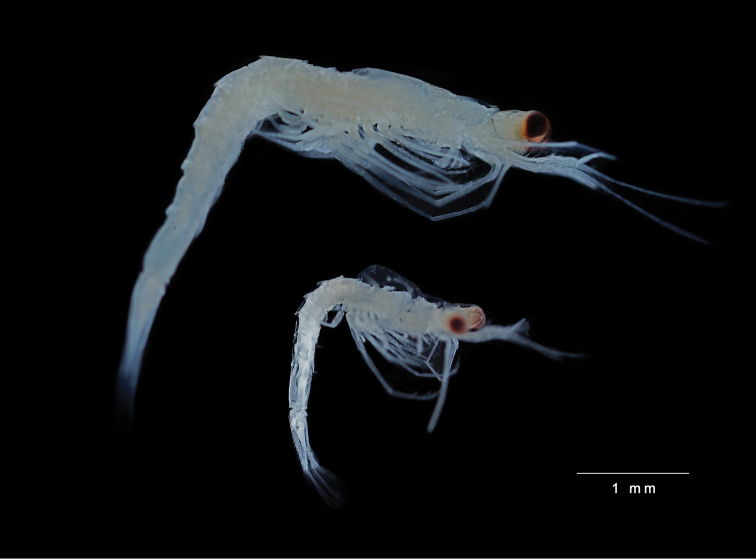
*Rhopalophthalmus longipes* Ii, 1964, **A** immature female, 6.5 mm, UKMMZ-1553 **B** juvenile, 2.4 mm, UKMMZ-1554, Pulau Tinggi, Malaysia.

*Carapace* short; anterior dorsal part of carapace between postorbital spines slightly produced, forming evenly rounded rostral plate; the postorbital spines sharp, supported by very short, feebly developed carina; antero-lateral angles of the carapace (“cheeks”) somewhat sinuous or slightly concave; posterior dorsal margin excavate, leaving the last two to three posterior thoracic somites exposed completely in dorsal view; cervical sulcus well marked dorsally and laterally around anterior one-third, nodules not present on the dorsal surface of carapace, just posterior to cervical groove in addition to posterior one.

**Figure 3.**
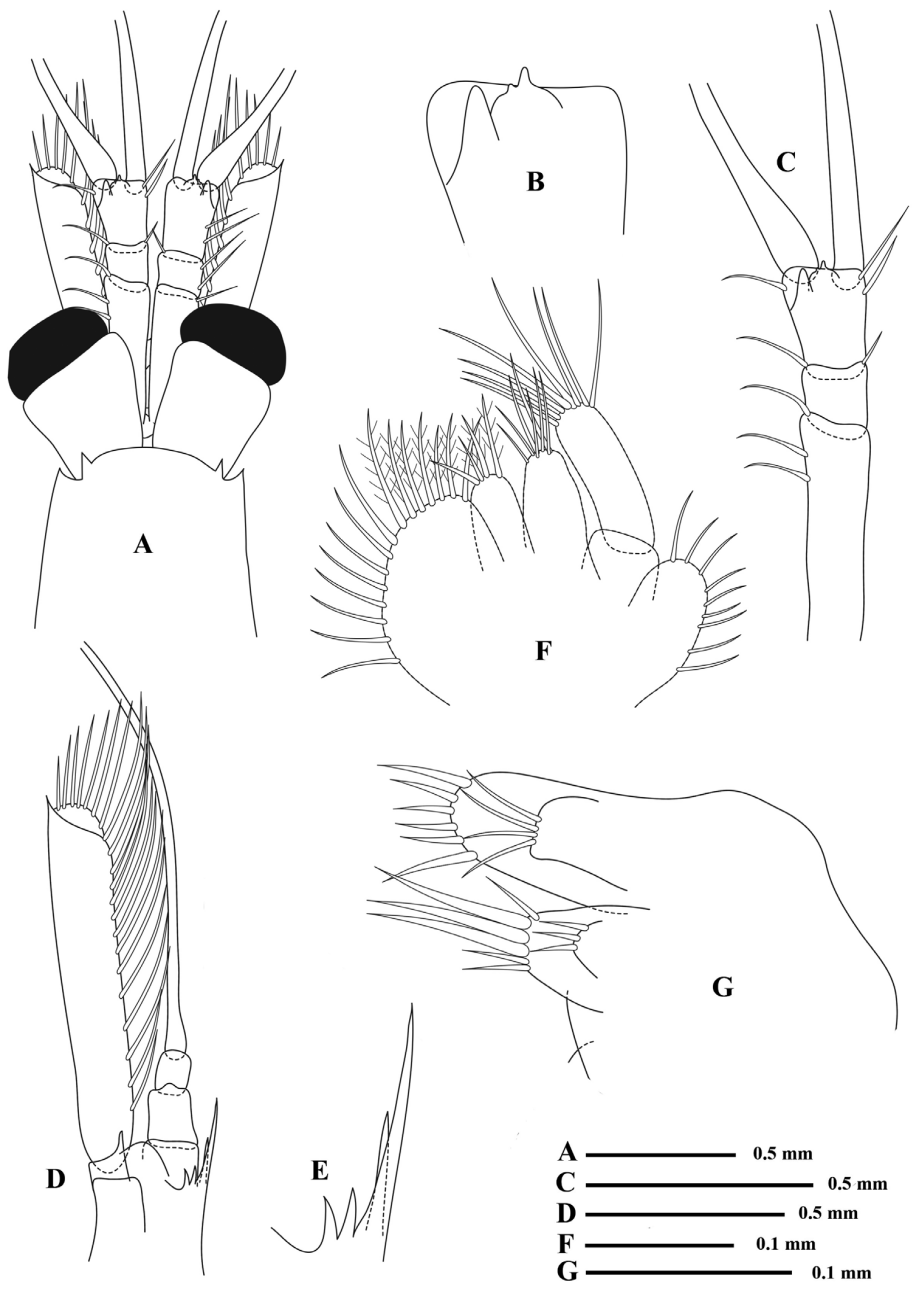
*Rhopalophthalmus longipes*; **A** anterior part of carapace **B** third segment of antennular peduncle **C** antennule **D** antenna and antenna scale **E** sympod spines of antenna **F** maxilla **G** maxillule.

*Eyes* large and globular, somewhat shorter than the first joint of antennular peduncle; cornea well pigmented; the whole eye, including the stalk, nearly 1 ½ times as long as broad, stalk nearly cylindrical, cornea occupying ⅓ of the eye and somewhat narrower than the distal end of the stalk (Fig. 3A).

*Antennular peduncle* somewhat slender, first segment of antennular peduncle 1 ⅓ times as long as the combined length of distal two segments, armed with several setae along lateral margin; second segment shortest, slightly shorter than wide; third segment longer than wide, with three moderate setae, distal outer corner produced into a triangular process under the base of the outer flagellum and a rounded process bearing two protrusions (Fig. 3B) apically near the middle distal end (Fig. 3C).

*Antennal scale* extending beyond the distal end of the antennular peduncle, approx. 6⅓–7 times as long as wide, the margins nearly parallel and equal width throughout; apex almost truncate; a distinct oblique suture marking off the small distal segment; disto-lateral spine slender, prominent and extending beyond the apex of the scale; sympod composed of four spines on the inner ventral face at the base of peduncle (Fig. 3E), two longer and two short spines, the most inner lateral spine around five times longer than the mesial one (Fig. 3D).

*Labrum* transverse, without process in front, mandibles with moveable lacinia thick, molar process thick, slightly produced, with teeth on the end, the palp moderately short, very feebly expanded. Maxilla (Fig. 3F) with the lobe from third segment deeply cleft, the palp elongated, the exopod rather small.

*Abdominal somites* smooth, second to fifth somites nearly sub-equal in length, first and sixth somites 1 ½ times as long as fifth one (Fig. 5A).

**Figure 4.**
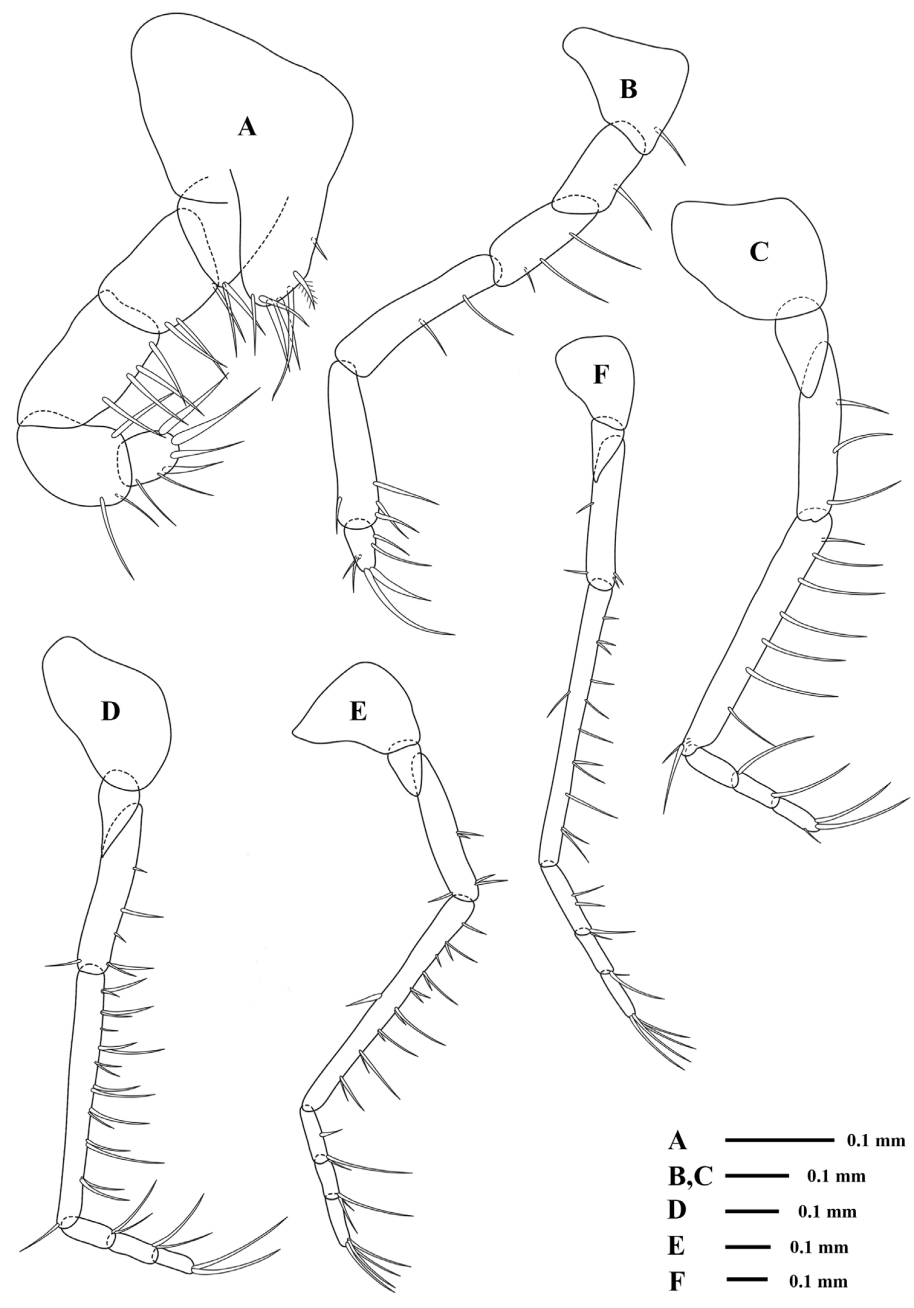
*Rhopalophthalmus longipes*; **A** endopod of first pereopod **B** endopod of second pereopod **C** endopod of third pereopod **D** endopod of fourth pereopod **E** endopod of fifth pereopod **F** endopod of sixth pereopod.

**Figure 5.**
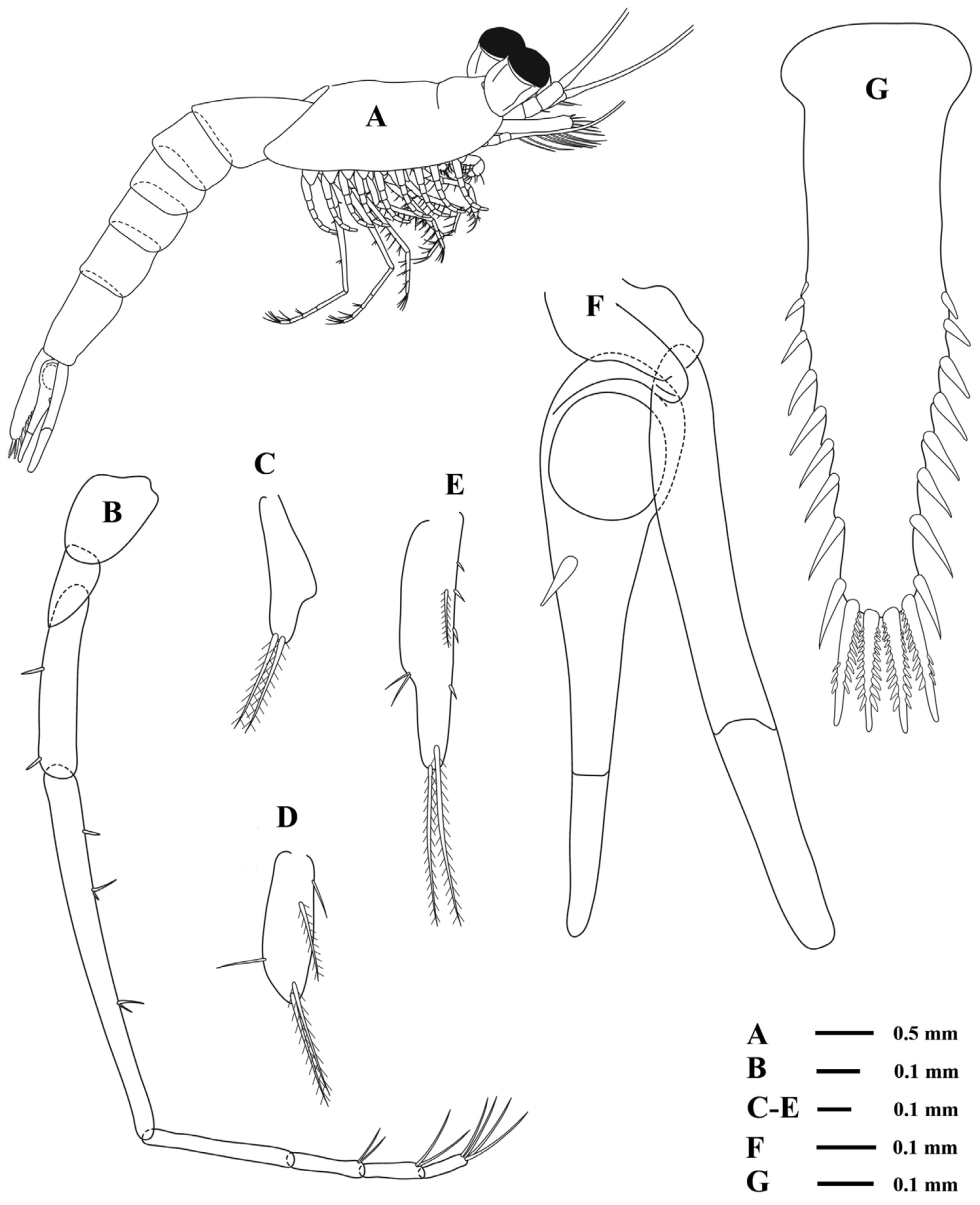
*Rhopalophthalmus longipes*; **A** immature female (lateral view) **B** endopod of seventh pereopod **C** first pleopod **D** third pleopod **E** fifth pleopod **F** right uropod **G** telson.

Endopods of *pereopods* (Figs 4A, B, C, D, E, F, 5B) slender and gradually increase in length posteriorly, remarkable in having proportionately long endopod particularly in seventh one; endopod of third to sixth pereopods similar in shape but length increasing posteriorly; endopod of third pereopod (Fig. 4C) slightly stouter than the fourth one, carpo-propodus divided into three articles, basal article sub-equal or slightly longer than the carpo-propodus; endopod of fourth to sixth pereopods (Figs 4D, 4E, 4F) having three-segmented carpo-propodus; endopod of seventh pereopod (Fig. 5B) longest, approx. 2½ times as long as the exopod, carpo-propodus divided into four articles, carpus noticeably long, as long as or longer than pereopod normally un-articulated, barely reaching mid-length of basal plate of exopod.

*Pleopods* un-articulated, length generally increasing on posterior somites but that on third pleopod somewhat short, comparable to first one (Figs 5C, 5D, 5E).

*Uropod* two-segmented in both endopod and exopod; endopod sub-equal in length with telson, proximal segment with a strong stout seta at the middle of the ventral inner margin, distal segment ¼ of the endopod in length; exopod with outer margin very fine setose, somewhat longer than endopod with distal segment ⅖ of the exopod in length and 10½ times as long as wide (Fig. 5F).

*Telson* comparatively narrow and slender, 4 ⅖ times as long as basal wide, nearly same length as the sixth abdominal somite, extends distinctly beyond the articulations of the uropod, abruptly constricted beyond the articulations of the uropod but not forming discernible waist, and hardly broadens to first ½ point with the lateral margins nearly parallel, in the next half gradually narrows distally with convex margins somewhat concave, and accordingly rather slightly broadens distally near the apex; distal half of the lateral margin armed with 7–9 strong spines, increasing in length posteriorly in the distal part but become again somewhat shorter towards the apex; apex narrowly rounded and armed with four extremely strong spines; the apical spines nearly equal in length with each other, ⅕ of the total length of the telson and furnished with secondary spinules, which are flattened like saw-teeth (Fig. 5G).

##### Type locality.

Shizuoka, Japan

##### Distribution.

Shizuoka, Nagasaki, Japan (Ii, 1964); Nansha Islands, the Spratlys (Wang and Liu, 1994); East China Sea (Wang and Liu, 1997); off Amami Island, south-western Japan; south-western part of South China Sea and western part of Timor Sea (Hanamura et al., 2011) and Pulau Tinggi, Johor, Malaysia (present study).

##### Remarks.

*Rhopalophthalmus longipes* was first described by Ii (1964) based on the specimens collected from Japan. This species can be easily distinguished from others species in having a very narrow telson and secondary spinules on the apical spines of telson. Another distinct character within the genus is the endopod of third to seventh thoracopods gradually increasing in length posteriorly and the seventh endopod of thoracopod being more than twice as long as the exopod.

*Rhopalophthalmus longipes* resembles *Rhopalophthalmus orientalis*, which was described by Tattersall (1957) from Japanese waters by having two long spines and two shorter spines at the antennal sympod and possessed peculiarly flattened teeth-saw like secondary spinules on the apical spines of telson. However, the seventh endopod of thoracopod in latter species is not as long as twice the length of exopod compared to *Rhopalophthalmus longipes* and the telson is conspicuously slender in the distal half compared to *Rhopalophthalmus orientalis*, which is moderately broad in distal half. In addition, *Rhopalophthalmus orientalis* has small triangular rostrum, which is not found in the *Rhopalophthalmus longipes*. *Rhopalophthalmus longipes* also shows resemblance to *Rhopalophthalmus terranatalis* O. Tattersall, 1957 collected from estuarine waters around the coasts of Natal from Richard’s Bay (on the east to Langebaan Bay of the south-west coast), but seventh thoracic endopod of the latter species has seven sub-segments instead of four sub-segments with an unusually elongated carpus in *Rhopalophthalmus longipes*.

The specimens found in this study exhibit some slight differences from the *Rhopalophthalmus longipes* of Hanamura et al. (2011) as the small nodules near the cervical sulcus of the carapace were absent. The specimens at hand also differ from the specimens described by Ii (1964) and Hanamura et al. (2011) by the combination of these characters; 1) presence of a triangular process under the base of the outer flagellum; 2) presence of a rounded process bearing two protrusions apically near the middle distal end of third segment of antennule peduncle; and 3) telson armed with only 7-8 moderately strong setae.

## Supplementary Material

XML Treatment for Rhopalophthalmus longipes
